# Oligosaccharide Substrate Preferences of Human Extracellular Sulfatase Sulf2 Using Liquid Chromatography-Mass Spectrometry Based Glycomics Approaches

**DOI:** 10.1371/journal.pone.0105143

**Published:** 2014-08-15

**Authors:** Yu Huang, Yang Mao, Jo Ann Buczek-Thomas, Matthew A. Nugent, Joseph Zaia

**Affiliations:** 1 Center for Biomedical Mass Spectrometry, Department of Biochemistry, Boston University School of Medicine, Boston, Massachusetts, United States of America; 2 Department of Biochemistry, Boston University School of Medicine, Boston, Massachusetts, United States of America; University of Patras, Greece

## Abstract

Sulfs are extracellular endosulfatases that selectively remove the 6-*O*-sulfate groups from cell surface heparan sulfate (HS) chain. By altering the sulfation at these particular sites, Sulfs function to remodel HS chains. As a result of the remodeling activity, HSulf2 regulates a multitude of cell-signaling events that depend on interactions between proteins and HS. Previous efforts to characterize the substrate specificity of human Sulfs (HSulfs) focused on the analysis of HS disaccharides and synthetic repeating units. In this study, we characterized the substrate preferences of human HSulf2 using HS oligosaccharides with various lengths and sulfation degrees from several naturally occurring HS sources by applying liquid chromatography mass spectrometry based glycomics methods. The results showed that HSulf2 preferentially digests highly sulfated HS oligosaccharides with zero acetyl groups and this preference is length dependent. In terms of length of oligosaccharides, HSulf2 digestion induced more sulfation decrease on DP6 (DP: degree of polymerization) compared to DP2, DP4 and DP8. In addition, the HSulf2 preferentially digests the oligosaccharide domain located at the non-reducing end (NRE) of the HS and heparin chain. In addition, the HSulf2 digestion products were altered only for specific isomers. HSulf2 treated NRE oligosaccharides also showed greater decrease in cell proliferation than those from internal domains of the HS chain. After further chromatographic separation, we identified the three most preferred unsaturated hexasaccharide for HSulf2.

## Introduction

Heparan sulfate (HS) is the most highly sulfated class of mammalian glycosaminoglycan polysaccharides.[1], [2] Through their ability to interact with various growth factors, chemokines, receptors and extracellular matrix molecules, HS is involved in biological processes including homeostasis, inflammation, angiogenesis, cell differentiation and proliferation.[3]–[5] Heparin is a highly sulfated form of HS expressed in granulated cells. [6], [7] It represents the most negatively charged biomolecule and is widely used as an anticoagulant drug. HS/heparin chains are biosynthesized in the Golgi apparatus as polysaccharides of (4GlcAβ1-4GlcNAcα1-) repeating disaccharide units that undergo a series of subsequent biosynthetic modification reactions including deacetylation, *N*-/*O*-sulfation and epimerization. The HS/heparin chain is attached to a core protein with a tetrasaccharide linker.[6]–[8] After its biosynthesis, mature HS chains are exposed to the cell surface and/or extracellular matrix environment and may undergo further processing by extracellular enzymes including mammalian heparanase and sulfatases. [9], [10]

Extracellular sulfatases (Sulf1 and Sulf2) catalyze the hydrolysis of the sulfate ester bond at the C6 position of GlcN residues of HS/heparin. [11]–[13] Sulf1 and Sulf2 share similar protein structures and show a degree of functional redundancy. [14]–[18] By specifically removing a subset of the 6-*O*-sulfate groups, Sulfs serve to modify the HS chains in various biological contexts. [16], [19]–[23] Sulfs are involved in alteration of the binding of HS to extracellular signaling molecules including glial cell-derived neurotrophic factor, bone morphogenetic protein, Sonic hedgehog homolog, fibroblast growth factor 2, vascular endothelial growth factor and transforming growth factor beta [12], [18], [24]–[31]. While Sulfs may function as positive regulator in certain signaling pathways, opposing effects were found for different pathways. [23], [32], [33] Increasing evidence from recent studies suggest Sulfs function as critical regulators in the pathogenesis of many cancers [30], [35]–[41]. In addition, PI-88, a polysulfonated phosphomannan polysaccharide preparation that has antiangiogenic and antimetastatic properties, was found to inhibit Sulfs. [34] Recent mRNA array analysis showed that genes corrected with both Sulfs were associated with signaling pathways including cell adhesion, extracellular matrix remodeling, blood coagulation and epithelial mesenchymal transition. Of the two enzymes, Sulf2 is more associated with neoplastic processes. [35] The long list of important growth factors and morphogens with which they interact, the growing number of signaling pathways involved, and the relevant physiological and pathophysiological states all implicate the important roles of Sulfs. Due to their roles in carcinogenesis, [30], [35]–[41] Sulfs represent attractive therapeutic target for cancer treatment. [38], [42]

A number of previous studies have characterized the HS context in which Sulfs process the 6-*O*-sulfation pattern. [11], [25], [43]–[48] Most of these relied on the analysis of fully depolymerized HS and found that disaccharides IdoA2S-GlcNS6S and GlcA-GlcNS6S are susceptible to Sulfs while IdoA-GlcNS6S is a less susceptible Sulf target [11]. Moreover, this preference for disaccharides was also found to be species-specific in that avian QSulfs differ from those of mammalian Sulfs. Other studies showed that the non-reducing end saturated disaccharides are more susceptible to Sulf2 digestion and implicated Sulf2 as a key regulator of the structure of the non-reducing end region. [45] Investigation of Sulfs substrates at the oligosaccharide level was performed by using HS extracted from *SULF* gene knock-out mice. [44] This work demonstrated that the 6-*O*-sulfate digestion pattern produced by murine Sulf1 was extensive and more distributed throughout the HS chain compared to its avian QSulf orthologues. However, due to reliance on heparin lyase III for HS depolymerization, Sulf digestion patterns were observed only for HS oligosaccharides not susceptible to this enzyme. In addition, genetic manipulation of *SULF* genes has been shown to alter the overall sulfation pattern by affecting the expression of other HS biosynthetic enzymes [47], thus making the assessment of these HS as Sulfs substrates less convincing. Synthetic oligosaccharides with repeating disaccharide or tetrasaccharide units were also utilized for determining the substrate specificity of Sulf2. [49] In this work, synthetic oligosaccharides corresponding to naturally occurring sequences confirmed previous studies from disaccharides analysis regarding preferred Sulf substrates.

In the present study, we aimed to extend the understanding of the substrate preference of human Sulf2 (HSulf2) and assess the effect of Sulf2 on certain biological processes. By taking advantage of the high throughput of liquid chromatography mass spectrometry based glycomics approaches [50], [51], we studied defined oligosaccharide with various lengths, sulfation/acetylation degrees and domain origins as substrates for HSulf2. The variety of these oligosaccharides digested from naturally occurring HS sources enabled us to examine a wide variety of saccharides for HSulf2 susceptibility. In addition, we studied the effect of Sulf2 digestion on HS oligosaccharides ability to support FGF-2 mediated cell proliferation and found a special role for the non-reducing end (NRE) region during HSulf2 digestion. Finally, we separated and identified the three most active unsaturated hexasaccharide substrates for HSulf2.

## Materials and Methods

### Materials

Porcine intestinal mucosa heparan sulfate was purchased from Celsus Laboratories, Inc. (Cincinnati, OH). Heparin lyase I, II and III from *Flavbacterium heparinum* were purchased from IBEX (Montreal, QC). Recombinant human sulfatase 2 was a generous gift from Shire Human Genetic Therapies (Cambridge, MA).

### HS Oligosaccharides Preparation

Digestion of porcine intestinal mucosa heparan sulfate (350 µg) was performed in a 1 ml solution system with 500 µl digestion buffer (100 mM NaCl, 20 mM Tris-HCl, 1 mM Ca(OAc)_2_, pH 7.4) at 37 °C. An aliquot of 50 mIU of a single heparin lyase enzyme (lyase I or lyase III) was added for overnight for complete digestion. The digestion was stopped by heating at 100 °C for 10 min. The digestion products were dried by centrifugal evaporation, reconstituted in water and purified/profiled using a Superdex™ peptide PC 3.2/30 column (GE healthcare). The column was equilibrated and operated using a 50 mM ammonium acetate buffer in 10% acetonitrile. The fraction corresponding to DP4 (DP: degree of polymerization), DP6 and DP8 oligosaccharides were collected for HSulf2 treatment and further MS analysis.

### Treatment of HS Oligosaccharides with HSulf2

HS oligosaccharide samples were dissolved in the Sulf digestion buffer (50 mM NaCl, 20 mM Tris, 1 mM MgCl_2_, pH 7.4) and an aliquot of HSulf2 in storage buffer (20 mM sodium phosphate, 500 mM NaCl, 10% glycerol, 0.5 mg/mL pefabloc, pH 7.0) was added. Another aliquot of HSulf2 was heat inactivated in 100 °C for 10 min for the control experiment. The experiment and control reactions were allowed to proceed overnight at 37 °C. Subsequently, the reaction was heat inactivated by boiling for 10 min.

### Amide-HILIC LC-MS Composition Analysis of HS Oligosaccharides

Each aliquot of about 5 to 10 pmol HSulf2 treated HS oligosaccharide samples and control samples was profiled for their composition using the makeup flow HPLC-chip based LC-MS method previously developed in our laboratory. [50] Briefly, the HPLC mobile phases were as follows: solvent A was 10% acetonitrile, 50 mM formic acid, pH 4.4 and solvent B was 95% acetonitrile, 5% solvent A. Samples were loaded onto the trapping column with a solvent composition of 75% to 85% B at 4 µL/min for a period of 10 min based on the length of the oligosaccharide. Afterwards, the trapping column was placed in-line with the analytical column and a gradient to 0% B was run over a period of 39 min at 200 nL/min. Following the gradient, the trapping column and analytical column were washed with 0% B for 10 min. The return to initial conditions was made over 10 min, followed by 10 min of equilibration. An extra 200 nL/min Makeup flow of acetonitrile was supplied during the entire run. The HPLC-chip system was on-line with an Agilent 6520 QTOF operating in the negative-ion mode.

### Size Exclusion Chromatography (SEC) LC-MS of HS saccharides

Heparin lyase digestion products ranging from disaccharide to tetrasaccharide, were directly analyzed using SEC-MS as described previously. [45], [52] Briefly, HS samples (∼100 pmol) were injected onto a Superdex Peptide PC 3.2/30 column (GE Biosciences, Piscataway, NJ) online with an Applied Biosystems/MDS Sciex QSTAR Pulsar Qq-TOF mass spectrometer operating in the negative-ion mode. The isocratic mobile phase contains 12.5 mM formic acid, titrated to pH 4.4 with ammonia, in 10% acetonitrile.

### Strong Anion Exchange Chromatography of HS oligosaccharide

Approximately 30 nmol of Sulf digestion products and control were loaded on to an IonPac AS7 column (4.6×250 mm, Thermo Scientific) using the method previously described. [44] Briefly, a Beckman Gold HPLC was running at 0.5 mL/min using a linear gradient as follows: mobile phase A, H_2_O (pH 3.5 by HCl); mobile phase B, 2 M NaCl (pH 3.5 by HCl); 1–10 min, 0%B, 10–70 min, 0–100%B; 85–90 min, 100–0%B. UV (232 nm) chromatographic peaks were collected and desalted for further analysis.

### BaF32 Cell Proliferation Assay

This method for accessing the cell proliferation of HS oligosaccharide mediated by FGF2 has been previously described. [53], [54] Briefly, BaF32 cells were firstly washed in medium lacking WEHI-3B conditioned medium were seeded onto Costar 96 well plates in the presence of 5 ng/ml FGF2. Dose-response plotting was generated using 0–150 pmol HS DP6 before and after HSulf2 digestion. Changes in cell proliferation were determined using MTT Cell Proliferation Assay (ATCC, Manassas, VA). After addition of MTT (3-(4,5-Dimethylthiazol-2-yl)-2,5-diphenyltetrazolium bromide) reagent, cells were incubated at 37 °C for 1 hour and the absorbance was measured at 570 nm 2 hours after stopping the reaction with addition of detergent. [54]

### Data Analysis and HS Oligosaccharide Composition Nomenclature

The HILIC LC-MS spectra were decovoluted using the combination of customized Decon2Ls/DeconTools Autoprocessor from Pacific Northwest National Laboratory and Progenesis (Nonlinear Dynamic Limited). The profiling of the HS compositions was achieved using in-house fitting programs. The composition coding of the HS and heparin is presented as [ΔHexA, HexA, GlcN, Ac, SO_3_] (ΔHexA: Δ^4,5^-unsaturated hexuronic acid; HexA: hexuronic acid; GlcN: glucosamine; Ac: acetyl group; SO_3_: sulfate), denoting the number of the corresponding residues.

## Results

### HSulf2 Treatment of Porcine Intestinal Mucosa HS Heparin Lyase I Oligosaccharides

Previous approaches for studying the substrates of the Sulfs relied primarily on disaccharide analysis. The resulting depolymerized disaccharides do not fully represent the context of HSulf2 enzyme activity. Recently, the Liu group has utilized relatively long chains of repeating HS disaccharide/tetrasaccharide units that were enzymatically synthesized from heparosan or bovine kidney HS as Sulf substrates. [49] Although insights on HSulf2 substrates specificity and implication in synthesizing anticoagulant HS were conclusive, the readout for the substrates specificity was still confined within the disaccharide level and was not directly related to oligosaccharide context for the enzyme. Here, we used naturally occurring HS oligosaccharide with various lengths and sulfation levels digested from different lyase to better represent the vast varieties of oligosaccharide domain structures present in HS. To generate structurally diverse HS oligosaccharides yet maintain the feasibility of analysis for HSulf2, we first used heparin lyase I to digest porcine intestine mucosa HS to obtain oligosaccharides with relatively high sulfation degrees.

**Figure 1:**
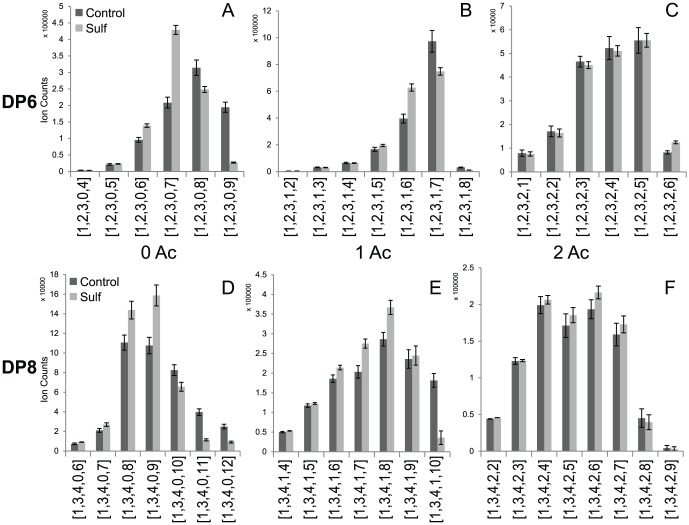
HS oligosaccharides composition profiles (from heparin lyase I digestion) before and after HSulf2 digestion. A, B and C show HS oligosaccharide DP6 with zero, one and two acetate groups separately. D, E and F show HS oligosaccharide DP8 with zero, one and two acetate groups separately. Error bar represents the standard deviation from triplicate LC-MS runs.

Digestion products were separated using a gel filtration column and DP6 to DP8 fractions were collected and used as the substrates for HSulf2. The compositions of the oligosaccharides before and after the HSulf2 treatment determined using HILIC-MS are shown in Fig. 1. For DP6 with zero acetate, the abundance of the two mostly highly sulfated compositions [1,2,3,0,9] and [1,2,3,0,8] dropped by 86% and 21%, respectively. These two HS compositions represent the most susceptible substrates for HSulf2. Considering the biosynthetic pathway and the specificity toward heparin lyase I, the most likely disaccharide component of this fully sulfated [1,2,3,0,9] are the repeating units of tri-sulfated disaccharides (IdoA2S-GlcNS6S). For [1,2,3,0,8], one di-sulfated disaccharide, presumably GlcA-GlcNS6S, exists along with two tri-sulfated units, consistent with previous results from disaccharide analysis. The observed decrease in abundance of [1,2,3,0,8] from the LC-MS data resulted from a combination of undigested [1,2,3,0,8] and the action of the sulfatase on [1,2,3,0,9] to produce new [1,2,3,0,8]. We therefore calculated the corrected decrease in abundance of [1,2,3,0,8] to be 48% due to HSulf2 activity (assuming one sulfate was removed by HSulf2 from [1,2,3,0,9]). The abundance of [1,2,3,0,7] increased approximately one fold and this was due primarily to the digestion products from [1,2,3,0,8] and [1,2,3,0,9], although it was also possible that [1,2,3,0,7] was also digested to a very minor extent. For the DP6 with one acetate, although the abundance of [1], [2], [3], [1,8] was low, it was the most susceptible substrate, decreasing in abundance by 64% after HSulf2 digestion. In comparison, the [1], [2], [3], [1,7], with compensation for sulfate loss from [1], [2], [3], [1,8], decreased in abundance by only 25%. For DP6 with 2 acetate group, the changes of the oligosaccharide composition abundances were not significant, indicating they are not substrates for HSulf2. For DP8, there was also the trend that those HS oligosaccharide compositions with zero acetate decreased more in abundance compared to those compositions with one acetate group. The most effective substrates are typically the most highly sulfated compositions in the particular series. For the DP8 with two acetate groups, the abundances before and after HSulf2 treatment were not changed significantly.

**Figure 2:**
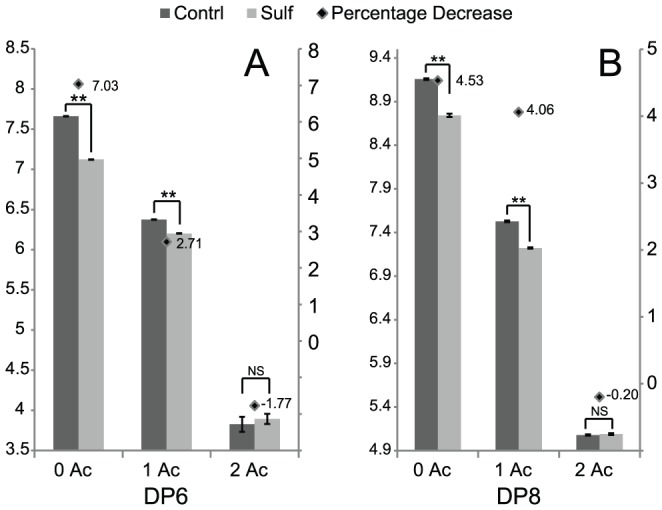
Changes of sulfate degree before and after HSulf2 digestion of HS oligosaccharides. A. HS DP6 sulfated changes for 0 Ac, 1 Ac and 2 Ac series. B. HS DP6 sulfated changes for 0 Ac, 1 Ac and 2 Ac series. Left Y-axis denotes the sulfation degree represented as the average sulfate number per disaccharide. Right Y-axis denotes the decrease in sulfate degree after HSulf2 digestion in percentage. Data significance was calculated by two-tailed Student's *t* test, with ** denoting p<0.01 and * denoting p<0.05. NS means difference not significant.

The abundance changes in terms of sulfation degree and acetate content from data in Fig. 1 is summarized in Fig. 2. It is obvious that sulfation degree of those DP6 with zero acetate group (2.55 sulfates per DP2) decrease by an average of 7% after HSulf2 treatment. However, those DP6 with one acetate group (2.12 sulfates per DP2) only decreased by 2.7% in sulfation degree upon HSulf2 treatment and no significant decrease was found for those with two acetate groups. In addition, the sulfation degree of DP6 with zero acetate decreases more than those of DP8 with zero acetate. In contrast, the DP8 with one acetate showed higher HSulf2 susceptibility than DP6 with one acetate, indicating that the effect of acetate was different as for the length of the oligosaccharides. After compensating for the changes by composition turnover, the degree of decrease abundance of each single composition was calculated and shown in Fig. S1 in File S1. It was obvious that highly sulfated, less acetylated compositions were decreased more in normalized abundance after HSulf2 digestion and thus were preferred substrates for HSulf2.

In addition to unsaturated even number of oligosaccharide originating from the internal region of HS the chains, we examined the saturated oligosaccharides and the odd number oligosaccharides from the NRE upon HSulf2 digestion, as shown in Figs. S2 and S3 in File S1. Although similar trend as unsaturated even number of HS oligosaccharide was observed, the extent of the decrease of the mostly highly sulfated compositions ([0,3,3,0,9], [0,3,4,0,10], and [0,3,4,1,9]) was more pronounced than that of the internal oligosaccharides, indicating that the non-reducing ends were more susceptible to HSulf2 digestion.

### HSulf2 Treatment of HS Oligosaccharides from Other Sources

In order to assess the HSulf2 substrate preferences more broadly, we incorporated various lengths of oligosaccharides from heparin lyase I digested heparin and heparin lyase III digested porcine intestinal mucosa HS for further investigation. Table 1 summarizes the profiles of the oligosaccharide compositions for DP4 to DP8 with zero or one acetate. The upper table shows the even number unsaturated oligosaccharides that originate from the internal part of the HS chains. The DP4 to DP6 oligosaccharides from heparin contain high degrees of sulfation ∼2.70 (represented as sulfate per DP2) and were susceptible to HSulf2 digestion. Specifically, heparin DP6 with zero acetate had the highest decreasing sulfation degree (7.6%) compared to DP4 and DP8. Heparin DP6 and DP8 with one acetate groups tended to be less susceptible to HSulf2 digestion in comparison with their zero acetyl group counterparts. However, HS oligosaccharides from lyase III digestion were less sulfated, with only 1.63 sulfate groups per DP2 on average for those with zero acetyl groups and even lower sulfation level for those that contain acetate groups (1.07–1.47 sulfate groups per DP2). After HSulf2 digestion, the sulfation levels decreased slightly with 0.3%∼1.2% decrease for HS oligosaccharide with zero acetyl group and only 0.3∼0.6% decreases for those with one acetyl group, indicating internal HS oligosaccharides from lyase III digestion were not favored by HSulf2.

**Table 1:** Original sulfation levels and sulfation changes of HS/heparin oligosaccharides after HSulf2 digestion.

				Unsaturated				
	Heparin oligosacc by lyase I				HS oligosacc by lyase III			
	0 Ac		1 Ac		0 Ac		1 Ac	
	Sulfation	Decrease	Sulfation	Decrease	Sulfation	Decrease	Sulfation	Decrease
**DP4**	2.70	4.1%	-	-	-	-	1.07	0.4%
**DP6**	2.70	7.6%	2.05	4.3%	1.63	1.2%	1.19	0.6%
**DP8**	2.50	5.6%	2.17	3.3%	1.63	0.3%	1.47	0.3%

				Saturated				
	Heparin oligosacc by lyase I				HS oligosacc by lyase III			
	0 Ac		1 Ac		0 Ac		1 Ac	
	Sulfation	Decrease	Sulfation	Decrease	Sulfation	Decrease	Sulfation	Decrease
**DP4**	2.65	2.4%	-	-	-	-	1.31	1.9%
**DP6**	2.73	5.0%	2.41	2.0%	2.26	8.40%	1.66	6.6%
**DP8**	2.53	3.6%	2.11	1.7%	2.12	1.40%	1.79	1.1%

The sulfation level is repsented as the aveage number of sulfates per disaccharide. Deceased percentage shows the sulfation level decrease in percentage after HSulf2 digestion.

The lower table of the Table 1 shows sulfation levels and changes upon HSulf2 digestion of the saturated oligosaccharides originated from the NRE. For both heparin and HS, these NRE oligosaccharides are all relatively highly sulfated with sulfation degree of 2.2∼2.7 sulfates per DP2 and almost all are susceptible to HSulf2 digestion. Interestingly, for saturated HS DP6 from lyase III digestion, the average sulfation level for zero acetate or one acetate group were 2.26 and 1.66 separately, and were significantly lower than those of heparin DP6 by lyase I. However, after HSulf2 digestion, they exerted 8.4% and 6.6% decrease of sulfation respectively, significantly higher than the same size oligosaccharide from heparin, despite the higher sulfation for the heparin oligosaccharides. Moreover, the observed 8.4% decrease in extent of sulfation for HS DP8 represents the highest decrease among all the oligosaccharides tested for HSulf2 digestion, indicating the NRE domain of the HS chain is the most susceptible domain for HSulf2 digestion.

In terms of size preference for HSulf2, for both unsaturated and saturated heparin and HS, DP6 tends to be more susceptible to digestion compared to DP4 and DP8. Also, direct digestion of DP2 by HSulf2 didn't show any changes of abundances (Fig. S4 in File S1), indicating that HSulf2 cannot work on oligosaccharides lower than DP4 and has a minimal preferred substrate of DP6. In light of the length of HSulf2 substrates preference, we used DP6 for further investigation into the activity of HSulf2.

### Effects of HSulf2 Digestion of HS DP6 on the Cell Proliferation

**Figure 3:**
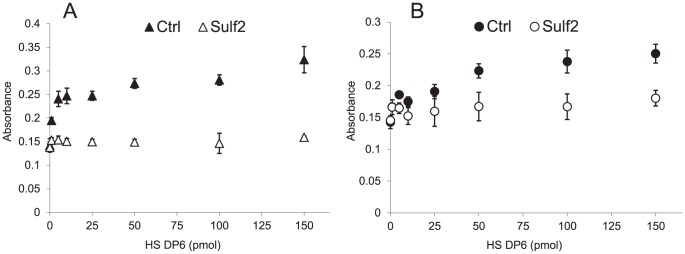
Dose-response plotting of BaF32 cell proliferation assays of two different HS DP6 sources upon HSulf2 digestion. A. HS DP6 from lyase III digestion. B. HS DP6 from lyase I digestion.

In order to determine the effects of HS sulfation decrease by HSulf2 activity on FGF2 medicated cell proliferation, we employed a miniaturized BaF32 cell proliferation assay that was applied previously [55]. HS DP6 produced using heparin lyase I with high sulfation level (2.12∼2.55 sulfates per DP2, Fig. 2) and HS DP6 produced using heparin lyase III with medium sulfation (1.19∼1.63 sulfates per DP2, Table 1, upper) were used to test the effects of HSulf2 on the FGF2 mediated cell proliferation. Full length bovine kidney HS was used as an activity control for the HSulf2 and the proliferation (Fig. S5 in File S1). The dose-response plotting of both HS DP6 samples before and after the HSulf2 digestion is shown in Fig. 3. It was obvious that HSulf2 treatment reduced the cell proliferation of both HS samples. However, despite the fact that the HS DP6 from lyase III (Fig. 3A) were not highly sulfated, showed only slight decreases in sulfation level (Table 1) and no significant decreases in the abundances of major components after HSulf2 digestion (LC-MS raw data of Table 1), we observed significantly decreased cell proliferation levels after HSulf2. These decreases were even more prominent than those of the highly sulfated HS oligosaccharides produced using heparin lyase I. It was not possible to chromatographically separate saturated oligosaccharides derived from the NRE from unsaturated DP derived from the HS chain interior. Nonetheless it is clear the susceptibility of sulfate removal by HSulf from NRE is responsible for prominent cell proliferation drop by HS DP6 from lyase III. Our results are therefore consistent with the conclusion that removals of sulfate groups from the NRE are likely to be the most the most physiologically significant effects of HSulf2 activity.

### Specific Isomeric Products from HSulf2 Digestion

**Figure 4:**
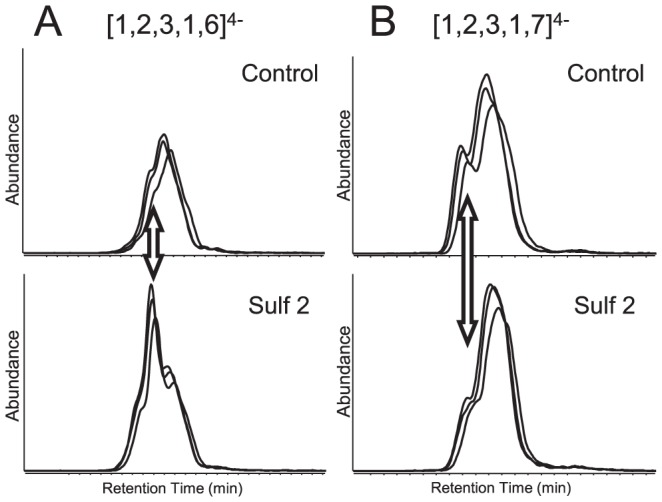
Extracted ion chromatograms (EICs) of -4 charged [1], [2], [3], [1,6] and [1], [2], [3], [1,7] before and after HSulf2 digestion from SEC-MS. EICs from three LC-MS runs were overlaid to the same scale and the double arrow shows the peak area that changed most upon HSulf2 digestion.

By using the glycomics approaches above, the composition information of the oligosaccharides were investigated in terms of sulfation degree and size. However, by modifying the only 6-*O*-sulfation, the digestion products also contains newly generated positional isomers with those resistant oligosaccharides. Specifically, the increased abundances of [1], [2], [3], [1,6] was due to the digestion of more highly sulfated compositions by HSulf2. Therefore, in addition to the original isomer s of [1], [2], [3], [1,6], the 6-*O*-desulfated [1], [2], [3], [1,7] and [1], [2], [3], [1,8] also contribute to the distribution of isomers of [1], [2], [3], [1,6] of the HSulf2 products, of which particular 6-*O*-sulfate groups are missing. This was confirmed by the partial chromatographic separation as shown in Fig. 4 for extracted ion chromatograms (EIC) of [1], [2], [3], [1,6] and [1], [2], [3], [1,7]. EICs before and after the treatment of HSulf2 digestion are shown in the same scale and EICs from triplicate runs were overlaid. After HSulf2 digestion, the increased abundance of [1], [2], [3], [1,6] was attributed to the increased peak of a particular isomer shown by the double arrow. In addition, we attribute the decreased abundance of the leading shoulder of [1], [2], [3], [1,7] to that of the isomeric component shown by the arrow. The EIC profiles show clearly that after HSulf2 treatment, only some of the saccharides were digested by HSulf2, specifically after removing the 6-*O*-sulfation. The resultant 6-*O*-sulfation depleted isomers represented the only increased abundance of the lower sulfate level oligosaccharides.

### Separation and the Composition of Unsaturated DP6 Substrates of HSulf2

**Figure 5:**
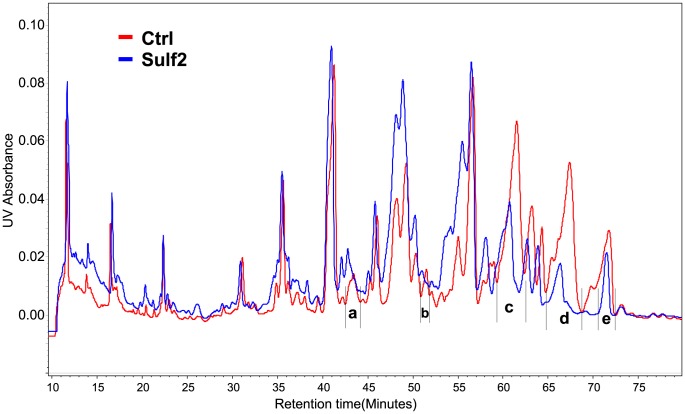
UV chromatograms of SAX separation of HS DP6 for control and HSulf2 digestion. Peak a∼e indicates major fractions that change upon Sulf digestion.

With the information from previous results, it is clear that only certain isomers from the oligosaccharides with highly sulfated/low acetate contain the most susceptible HSulf2 substrates. In order to investigate these oligosaccharides, we used high resolution strong anion exchange (SAX) chromatography for the further separation and analysis of the oligosaccharides treated by HSulf2. We used HS oligosaccharides DP6 from heparin lyase I digestion for this purpose since they were highly sulfated and proven susceptible to HSulf2 (Fig. 1). Moreover, the abundant susceptible oligosaccharides were in unsaturated form, allowing sensitive and facile detection of the HS oligosaccharides by UV absorbance. The UV chromatograms of the SAX separated HS DP6 before and after HSulf2 treatment are overlaid in Fig. 5. After the HSulf2 digestion, the UV abundance of the peaks from the mostly sulfated region (60–73 min) decreased and the abundances of the corresponding products were increased from 42–57 min. Peaks with abundance changes were collected and desalted for mass spectrometry analysis. For those peaks that decreased their abundance after HSulf2 digestion (**b**, **c**, **d** and **e**) the HS oligosaccharide composition were confirmed as shown in Table 2. Interestingly, although peak **e** contained the most highly sulfated composition [1,2,3,0,9], the abundance change was less than peak **d**, mainly composed of [1,2,3,0,8]. Also, although peaks **c** and **e** contained [1], [2], [3], [1,7], they differed in isomeric structures and thus responded differently to HSulf2 digestion. However, these results also demonstrated the necessity of highly sulfated saccharide units, presumably Glc2S-GlcNS6S, within the of HSulf2 substrate oligosaccharides.

**Table 2:** Composition profiling of the peaks from SAX separation with most abundance changes after HSulf2 digestion.

Peak	Composition
**a**	[1], [2], [2,1,4], [1], [2], [3], [1,5], [1,2,3,0,6], [0,3,3,0,7]
**b**	[1], [2], [3], [1,6]
**c**	[1], [2], [3], [1,7], [1,2,3,0,8]
**d**	[1,2,3,0,8]
**e**	[1], [2], [3], [1,7], [1,2,3,0,9]

Peaks **a∼e** represent chromatographic peaks from Fig. 5.

## Discussion

HS extracellular remodeling enzymes, namely heparanase and Sulfs, have increasingly drawn the attention of the cancer pathology and drug development communities. While oligosaccharide mimetic drugs that target heparanase have been developed, drug candidates for Sulfs remain underdeveloped. [42] In order for the development of such drugs and for basic understanding the enzymatic properties of Sulfs, it is necessary to determine the structure of HS Sulf substrates. However, primarily due to the heterogeneity of HS, analytical approaches have focused primarily on depolymerized HS disaccharides. [45], [48]. By looking at the abundance changes of disaccharides from Sulf-treated intact HS chains, it was found that the 6-*O*-sulfation from tri-sulfated IdoA2S-GlcNS6S was the most affected from various studies. In addition, disaccharide analysis effectively profiles the overall sulfation decrease caused by Sulf digestion. By using LC-MS based detection methods, it was possible to differentiate the difference response of disaccharides from internal region of chain or from the non-reducing end region of the chain. However, the HS context on which Sulfs was not fully revealed from disaccharide studies. The application of synthetic HS using repeating disaccharides or tetrasaccharide units greatly helped the substrate characterization the Sulfs. In an oligosaccharide context, it was concluded that repeating units of IdoA2S-GlcNS6S and in the presence of GlcA-GlcNS6S units has the most activity for Sulf2 compared to other 6S containing repeating units. However, these repeating units might only reside in a few domains in the naturally occurring HS chains, compared with heparin, in which such units are present in abundance. Naturally existing oligosaccharides extracted from *SULF*-null mice was also employed for substrate identification of Sulfs. However, mainly due to the fact the alteration of *SULF* would also affect other HS biosynthetic enzymes, thereby changing the overall sulfation pattern of HS chain [47], the results from this analysis were only partly consistent with previous disaccharide analysis results and did not adequately represent the oligosaccharide context recognized by Sulfs.

Attempts to analyze oligosaccharides digested from HSulf2 treated full length HS chains previously in our lab have encountered issues of unpredicted sulfates patterns from the composition profiling data that resulted from changes in the heparin lyases digestion pattern produced after by the treatment of HSulf2.

In light of the facts above, in this study, we started our investigation by using the HS/heparin oligosaccharides purified from natural sources to study the substrate preference of HSulf2. The oligosaccharides chosen were of various lengths (DP2 to DP8), sulfation and acetylation levels and from both internal chain and the non-reducing end region of the chain in order to represent the range of substrates encountered by Sulfs in the physiological environment. Using the oligosaccharide as substrates for Sulf2, we found that Sulf2 digested DP6 most effectively *in vitro*; DP2 alone, albeit tri-sulfated, was not acted upon by HSulf2; DP4 was digested to a limited extent. The optimal DP6 substrates for Sulf2 were highly sulfated, not containing acetyl groups, suggesting the repeating tri-sulfated DP2 is required as a triplet for the maximum processing by HSulf2. By comparison, DP8 averaging 3 sulfates per DP2 showed moderate sulfation decrease by HSulf2 digestion. Overall, considering the effect of HSulf2 on DP2 to DP8 and on longer chains, the data support the conclusion that HSulf2 acts preferentially on HS domains with repeating tri-sulfated disaccharides that contain 6-*O*-sulfation, and that the minimum length of these HS moieties was at least DP4 with the optimal size of DP6.

The results from the NRE oligosaccharides by Sulf2 also confirmed the previous disaccharides analysis that NRE domain was more susceptible to Sulf2 digestion and we extended the conclusion to oligosaccharide level. Moreover, BaF32 cell assays suggested that the effect of HSulf2 activity on cell proliferation was due to a greater extent to removal of 6-*O*-sulfate from NRE domains than from interior domains, further supporting the conclusion that NRE domain are critical for FGF2 binding [54] and the biological effect of the binding can be effetely regulated by Sulf2.

Direct and high throughput analysis of HS oligosaccharides has greatly improved after the extensive application of LC-MS based glycomics methods. By using HILIC LC-MS, we profiled HS compositions before and after Sulf2 digestion and observed changes in the partially resolved isomeric distributions resulting from HSulf2 digestion. Using further separation of these highly sulfated oligosaccharides we identified HS SAX peaks that have the highest susceptibilities to Sulf2.

In addition, primarily due to the extreme fragility of the sulfate modification and the complexity and variety in structure, methods for detailed structural characterization of HS oligosaccharides from multilevel separation were quite limited. Although tandem MS based methods are being developed as a sensitive and high throughput method for HS oligosaccharides; nevertheless, methods for very highly sulfated oligosaccharide, such as Sulf2 substrates, still need further development.

## Supporting Information

File S1 Contains the following files: **Figure S1. Normalized decreases for each HS composition assuming one sulfate removal turnover by HSulf2 from** **Fig. 1** **.** A. DP6 Oligosaccharides, B, DP8 oligosaccharides. **Figure S2. Non-reducing end oligosaccharide (from heparin lyase I digestion) profiles before and after HSulf2 digestion.** A, DP6 with zero acetyl groups, B, DP6 with one acetyl group, C, DP8 with zero acetyl groups, D, DP8 with one acetyl group. **Figure S3. Odd number DP7 profiles (from heparin lyase I digestion) before and after HSulf2 digestion.** A, B and C show DP7 with zero, one, and two acetyl group receptively. **Figure S4. EICs of 6-** ***O*** **-sulfate containing disaccharides before and after HSulf2 digestion.** **Figure S5. Controls for BaF32 cell proliferation assay.** A. Dose-response plotting of long chain bovine kidney heparan sulfate before and after HSulf2 digestion. B. Reponses of control for the cell proliferation assay.
(DOCX)
