# Supplementary material for: Oligosaccharide Substrate Preferences of Human Extracellular Sulfatase Sulf2 Using Liquid Chromatography-Mass Spectrometry Based Glycomics Approaches
Source: PLoS One. 2014 Aug 15;9(8):e105143. doi: 10.1371/journal.pone.0105143 (PMC4134258; doi:10.1371/journal.pone.0105143)

**Supporting Information for:**

**Oligosaccharide Substrate Preferences of Human Extracellular Sulfatase Sulf2 using Liquid Chromatography-Mass Spectrometry Based Glycomics Approaches**

Yu Huang^1^, Yang Mao^1^, Jo Ann Buczek-Thomas^2^, Matthew A. Nugent^2^, Joseph Zaia^1*^

^1^Center for Biomedical Mass Spectrometry, Department of Biochemistry, Boston University School of Medicine, Boston, MA 02118

^2^Department of Biochemistry, Boston University School of Medicine, Boston, MA 02118

*To whom correspondence should be addressed.

Address:

Center for Biomedical Mass Spectrometry, Department of Biochemistry, Boston University School of Medicine, 670 Albany Street, Room 509, Boston, MA 02118.

Tel: 617-638-6762.

Fax: 617-638-6761.

E-mail: jzaia@bu.edu.

**Figure S1**. Normalized decreases for each HS composition assuming one sulfate removal turnover by HSulf2 from Fig. 1. A. DP6 Oligosaccharides, B, DP8 oligosaccharides.


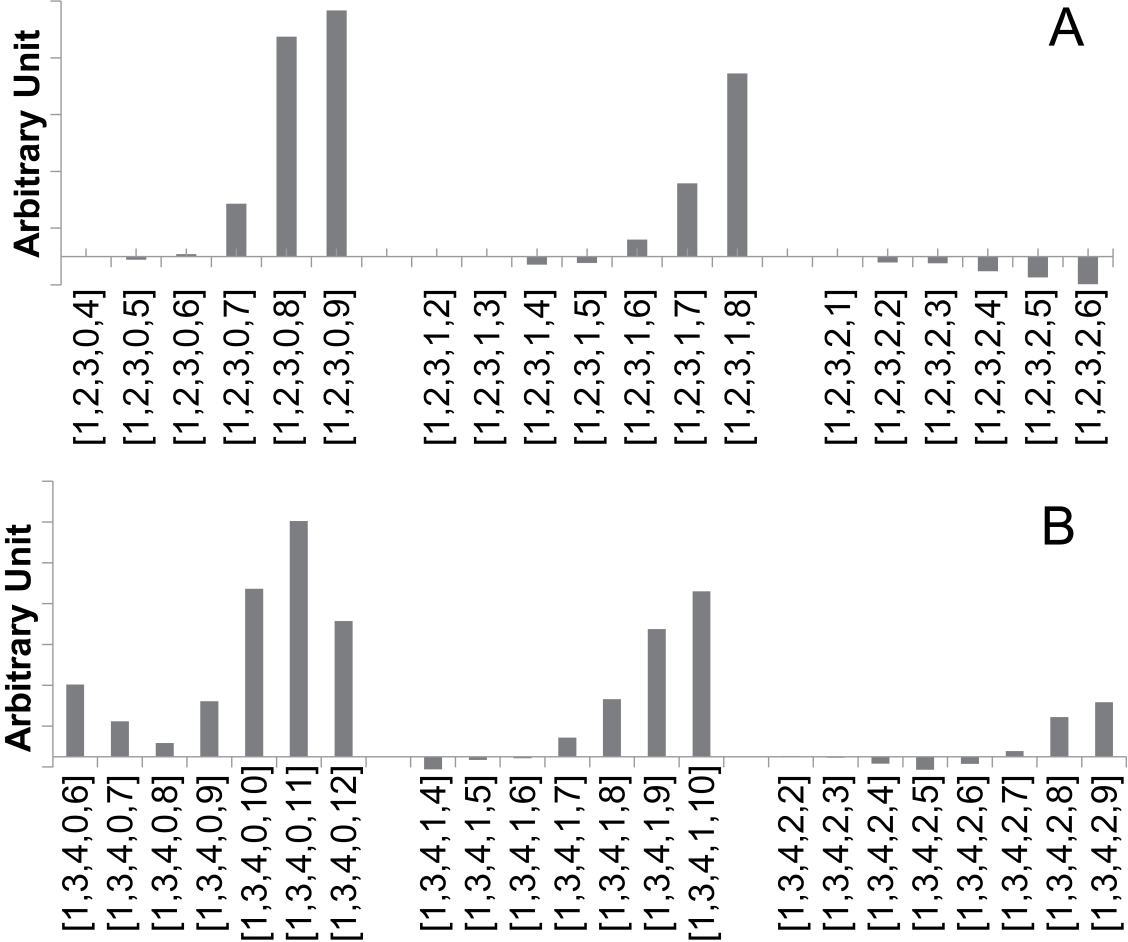


**Figure S2.** Non-reducing end oligosaccharide (from heparin lyase I digestion) profiles before and after HSulf2 digestion**.** A, DP6 with zero acetyl groups, B, DP6 with one acetyl group, C, DP8 with zero acetyl groups, D, DP8 with one acetyl group.


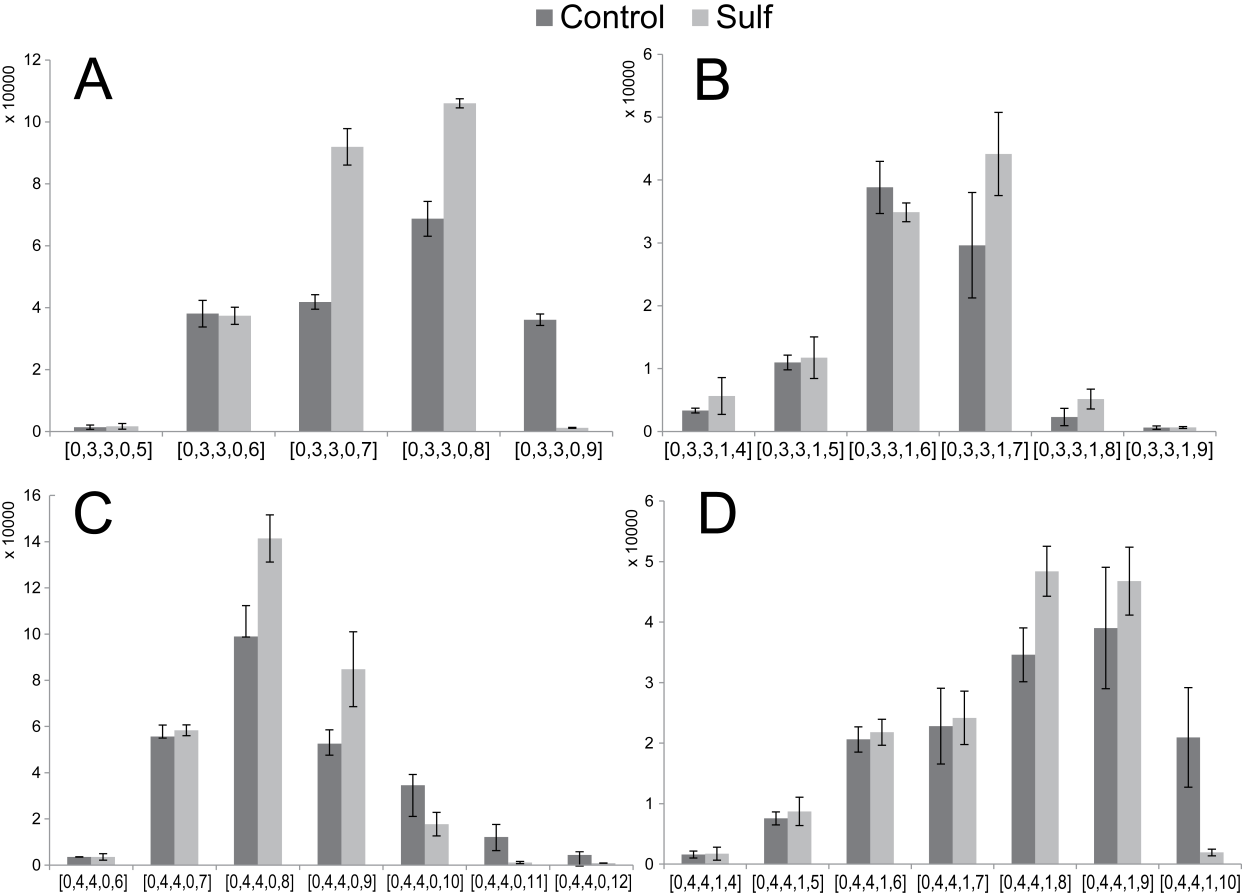


**Figure S3**. Odd number DP7 profiles (from heparin lyase I digestion) before and after HSulf2 digestion. A, B and C show DP7 with zero, one, and two acetyl group receptively.


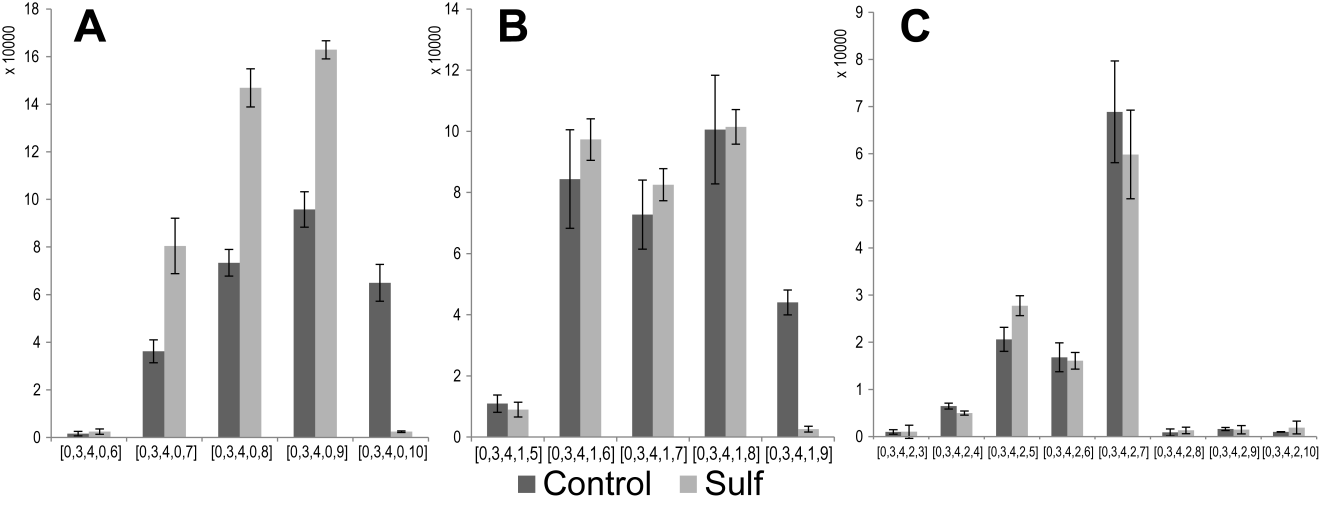


**Figure S4**. EICs of 6-*O*-sulfate containing disaccharides before and after HSulf2 digestion.


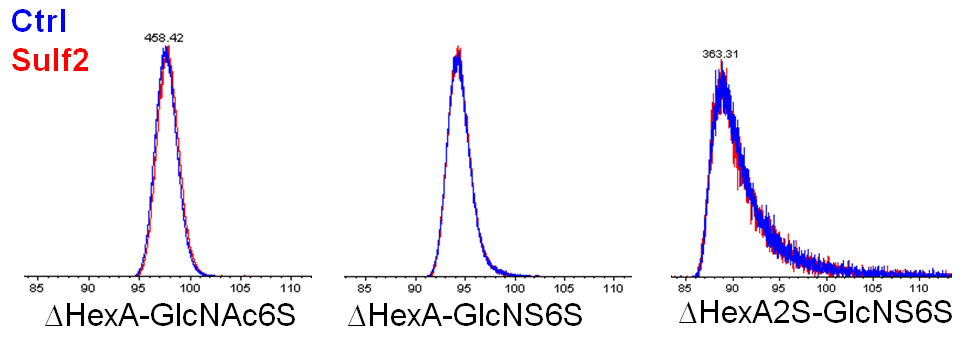


**Figure S5. Controls for BaF32 cell proliferation assay.** A. Dose-response plotting of long chain bovine kidney heparan sulfate before and after HSulf2 digestion. B. Reponses of control for the cell proliferation assay.


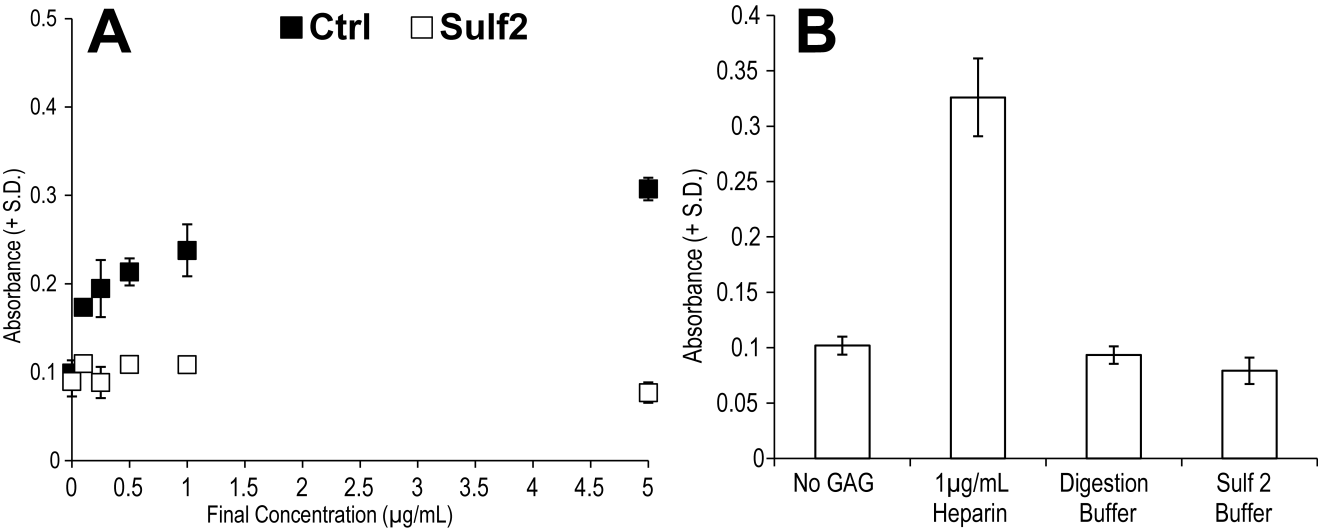

Supplement: File S1 — Contains the following files: Figure S1. Normalized decreases for each HS composition assuming one sulfate removal turnover by HSulf2 from Fig. 1 . A. DP6 Oligosaccharides, B, DP8 oligosaccharides. Figure S2. Non-reducing end oligosaccharide (from heparin lyase I digestion) profiles before and after HSulf2 digestion. A, DP6 with zero acetyl groups, B, DP6 with one acetyl group, C, DP8 with zero acetyl groups, D, DP8 with one acetyl group. Figure S3. Odd number DP7 profiles (from heparin lyase I digestion) before and after HSulf2 digestion. A, B and C show DP7 with zero, one, and two acetyl group receptively. Figure S4. EICs of 6- O -sulfate containing disaccharides before and after HSulf2 digestion. Figure S5. Controls for BaF32 cell proliferation assay. A. Dose-response plotting of long chain bovine kidney heparan sulfate before and after HSulf2 digestion. B. Reponses of control for the cell proliferation assay. (DOCX) [file pone.0105143.s001.docx]
